# Assessing the Impact of Aviation Emissions on Air Quality at a Regional Greek Airport Using Machine Learning

**DOI:** 10.3390/toxics13030217

**Published:** 2025-03-16

**Authors:** Christos Stefanis, Ioannis Manisalidis, Elisavet Stavropoulou, Agathangelos Stavropoulos, Christina Tsigalou, Chrysoula (Chrysa) Voidarou, Theodoros C. Constantinidis, Eugenia Bezirtzoglou

**Affiliations:** 1 Laboratory of Hygiene and Environmental Protection, Faculty of Medicine, Democritus University of Thrace, 68100 Alexandroupolis, Greece; giannismanisal@gmail.com (I.M.); elisabeth.stavropoulou@gmail.com (E.S.); ctsigalo@med.duth.gr (C.T.); tconstan@med.duth.gr (T.C.C.); empezirt@yahoo.gr (E.B.); 2 Delphis S.A., 14564 Kifisia, Greece; 3 School of Social and Political Sciences, University of Glasgow, Glasgow G12 8QQ, UK; angelostavrop@gmail.com; 4 Department of Agriculture, School of Agriculture, University of Ioannina, 47100 Arta, Greece; xvoidarou@uoi.gr

**Keywords:** air pollution, environment, health, airport, transport, public health, gas emission, machine learning models

## Abstract

Aviation emissions significantly impact air quality, contributing to environmental degradation and public health risks. This study aims to assess the impact of aviation-related emissions on air quality at Alexandroupolis Regional Airport, Greece, and evaluate the role of meteorological factors in pollution dispersion. Using machine learning models, we analyzed emissions data, including CO_2_, NOx, CO, HC, SOx, PM_2.5_, fuel consumption, and meteorological parameters from 2019–2020. Results indicate that NOx and CO_2_ emissions showed the highest correlation with air traffic volume and fuel consumption (R = 0.63 and 0.67, respectively). Bayesian Linear Regression and Linear Regression emerged as the most accurate models, achieving an R^2^ value of 0.96 and 0.97, respectively, for predicting PM_2.5_ concentrations. Meteorological factors had a moderate influence, with precipitation negatively correlated with PM_2.5_ (−0.03), while temperature and wind speed showed limited effects on emissions. A significant decline in aviation emissions was observed in 2020, with CO_2_ emissions decreasing by 28.1%, NOx by 26.5%, and PM_2.5_ by 35.4% compared to 2019, reflecting the impact of COVID-19 travel restrictions. Carbon dioxide had the most extensive percentage distribution, accounting for 75.5% of total emissions, followed by fuels, which accounted for 24%, and the remaining pollutants, such as NOx, CO, HC, SOx, and PM_2.5_, had more minor impacts. These findings highlight the need for optimized air quality management at regional airports, integrating machine learning for predictive monitoring and supporting policy interventions to mitigate aviation-related pollution.

## 1. Introduction

The surge in global economic growth has undeniably bolstered various sectors; however, this prosperity has been accompanied by a grave environmental repercussion—escalating concentrations of pollutants in the atmosphere. Human activities associated with urbanization, industrialization, and economic development have significantly contributed to this burgeoning ecological crisis. While these advancements have propelled society forward, they have concurrently triggered the production of harmful pollutants, adversely impacting the environment and human health. The resulting air pollution has emerged as a multifaceted global concern, encompassing social, economic, political, and legislative dimensions [1,2].

In the United States, the acknowledgement of the pressing nature of air quality concerns is evident in the mandate for the US Environmental Protection Agency (EPA) to reassess the National Ambient Air Quality Standards (NAAQS) every five years. This regulatory obligation underscores recognizing the dynamic nature of air pollution challenges and the necessity for adaptive strategies to mitigate its adverse effects. Similarly, in Europe, air pollution stands as a significant threat to environmental health, precipitating respiratory ailments, cardiovascular diseases, and premature deaths among populations. The staggering statistics depicting the correlation between morbidity and mortality rates and air pollution are alarming. Approximately nine million deaths annually are linked [3,4,5,6,7].

WHO figures show that most of the world’s population (99%) inhales air that exceeds setting guideline limits containing high levels of pollutants [8]. Global air pollution has increased by 8% from 2008 to 2013, with low- and middle-income countries showing the highest urban air pollution levels [8]. Nevertheless, pollution levels in some European cities exceed the limit values for pollutant concentrations [6]. Emissions through transport, industrial facilities, fires, and storms due to climate issues contribute to environmental degradation and impact public and individual health [1,7].

The activity of the transport sector emits air pollutants and increases levels of air pollution [1]. There is mounting evidence that emissions from aviation grow faster than any other mode of transport [2]. Aircraft are releasing carbon dioxide (CO_2_), carbon monoxide (CO), hydrocarbons (HC), nitrogen oxides (NO_x_), suspended particulate matter (PM), and sulfur oxides (SO_x_) [1,2]. Aviation emissions contribute as much to the global climate change [1]. However, air pollution is associated with numerous adverse health effects and many diseases [1].

COVID-19 restrictive measures were imposed internationally in the transport field to limit the spread of the virus. During this pandemic, restrictions and limited anthropogenic activities impacted the gloomy picture of air quality. Many studies were produced on this matter, reporting the reduction in air pollution during the pandemic due to the abovementioned measures [9,10]. In this vein, nitrogen oxide (NO_x_) concentrations and particle concentrations (PM_2.5_, PM_10_) were significantly reduced, while ground-level ozone (O_3_) levels rose [11,12]. Carbon monoxide (CO), as well as sulfur dioxide (SO_2_), showed abatement during the restriction period, but it has not been steady [13,14].

However, population-limited mobility and social distancing policies led to a downward trend in COVID-19 cases due to the measures taken [10]. Evaluating the aftermath of the COVID-19 pandemic on society and the economy is essential to providing responses and adapting governmental measures and policies to be applied [15]. Thus, the United Nations globally engaged its 131 country teams serving 162 countries to support governments and develop effective public health preparedness and responsiveness policies against the COVID-19 pandemic [15].

Still, socio-economic changes are observed related to gender inequalities, as women’s work increased due to both childcare as well as remote professional work at home [16]. Vulnerable groups in society have been strongly affected by the COVID-19 pandemic [17] following a study by NIVEL (Netherlands Institute for Health Services Research). The study is based on records from general practitioners and data collected from the Statistics Netherlands (CBS) organization. Low-income families or disadvantaged social groups appeared to be more vulnerable. Also, mental health seems to be seriously affected in several population groups as well as in people having pre-existing mental health problems [17,18]. Subsequently, according to the WHO (World Health Organization), the COVID-19 pandemic caused discriminatory behavior due to the social stigma against several disadvantaged ethnic groups [19] and people affected by the SARS-CoV-2 virus.

The selection of the given airport was based on the traffic, configuration, and area of the airport, as well as the variety of aircraft types operating at the airport. It will also be compared with prevailing environmental conditions, air pollution, regional development, and public health policy-making. Although various prediction models have been proposed by scientists in the field [20], there is still a need for more accurate models to develop effective prevention and control strategies in cases where threshold values rise to unacceptable levels for public health.

Having calculated the pollutant emissions, obtaining an image of their concentration in the areas of interest will be appropriate in two ways: by atmospheric dispersion calculation models and on-site measurements. Thus, we can have snapshots of the concentration of pollutants in the atmosphere at a specific location and time.

As stated previously, our study aims to record the current air pollution in the airport and assess the factors that influence the existing management model, intending to optimize it through a decision support system. While there is a wealth of research on air pollution, there needs to be more information on air pollution issued by airports. The existing studies in our country have mainly focused on “Eleftherios Venizelos”, the largest airport in Greece, which is closer to the data of a standard European airport. The lack of data and data recording in the regional airports, especially those in Eastern Macedonia and Thrace, aroused our interest in the present study. However, the restrictive policy due to the pandemic offers us an ideal model for comparative studies of the impact of pollution on air quality levels.

To summarize, this study aims to assess the air pollution at Alexandroupolis Regional airport in Greece and the influence of meteorological parameters on the dispersion of pollutants related to air transport operations. Furthermore, this study applies machine learning techniques to develop a methodological approach for predicting air pollutants and identifying critical environmental conditions that affect air pollution. Specifically, it will be assessed whether aviation emissions contribute significantly to local air pollution, with NOx, CO_2_, and PM_2.5_ being the dominant pollutants. Furthermore, we assume that meteorological conditions may impact the concentration levels of contaminants. Lastly, we present various machine learning models intending to increase the predictive ability for estimating pollutant levels, offering a valuable tool for air quality management, mainly at regional airports.

## 2. Materials and Methods

**Figure 1:**
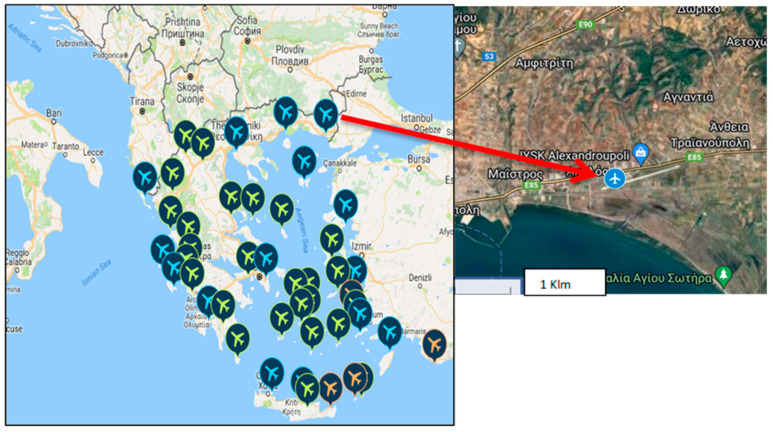
Study area—Alexandroupolis airport, Greece.

As we stated previously, our interest was focused on a study of air traffic in a regional Greek airport of Eastern Macedonia and Thrace, which is the airport of Alexandroupolis (Figure 1).

The Alexandroupolis “Democritus” civil airport is approximately 7.0 km east of Alexandroupolis in Evros Prefecture, Thrace (Northeastern Greece). The airport pays tribute to the ancient atomic philosopher Democritus, who hails from Avdira near Xanthi in Thrace.

Completed in 2011, the airport comprises terminal and administrative buildings covering over 8500 m^2^. Its coordinates place it at Latitude: 40°51′21″ North and Longitude: 25°57′22″ East. It comprises one terminal building, an administration building, a control tower, and a fire brigade station. At the same time, it holds a Category VI (6) rating for Airport Fire Fighting, providing four (4) parking positions tailored for medium-sized aircraft (http://www.ypa.gr/en/our-airports/kratikos-aerolimenas-alejandroypolhs-dhmokritos-kaald, accessed on 1 December 2023).

Our study was conducted from January 2019 to December 2020. Due to the containment measures, the impact of aircraft pollutants during the COVID-19 global pandemic, as a reduction in flight numbers, was registered. The Hellenic Civil Aviation Authority (CAA) collected all air traffic and fleet composition data.

Air pollutants from aircraft operations disperse based on several meteorological factors, including wind speed, temperature, and atmospheric stability. The dispersion patterns determine the extent to which pollutants such as NOx, CO, SOx, and PM_2.5_ reach populated areas near airports. Wind direction and speed significantly affect the transport of contaminants, while temperature inversions can trap emissions near the ground, leading to higher exposure levels in nearby communities. Manisalidis (2023) highlights that exposure to these pollutants is directly linked to respiratory and cardiovascular diseases, increased hospital admissions, and long-term health complications [21].

The emissions have been calculated using each aircraft’s emission factors, following the standard LTO emission factor methodology and the analytical methodology incorporated in the *EMEP/EEA Air Pollutant Emission Inventory Guidebook*, which includes emissions released at ground level and up to an altitude of 3000 feet, following the International Civil Aviation Organization (ICAO) guidelines. Specifically, pollutants such as NOx, CO, and PM_2.5_ were primarily assessed at ground level, where aircraft taxiing, takeoff, and landing emissions occur. However, some dispersion of pollutants into the lower atmosphere is expected, influenced by meteorological conditions such as wind speed, precipitation, temperature, and atmospheric stability [1]. By incorporating these factors, the study comprehensively assesses aviation emissions and their potential impact on local air quality.

Briefly, the emissions in this study were calculated using each aircraft’s emission factors according to the standard LTO emission factor methodology and the methodological approach described in the *EMEP/EEA Air Pollutant Emission Inventory Guidebook* [22]. The total emissions *E_m,a,p,I_* of a given pollutant *p* from aircraft type *I* at airport *a* over a specific period *T* were estimated using the simplified approach:

*E_m,a,p,I_* = 10^−6^ × *EF_p,i_* × Δ*_a,i_*

where:

- *E_m,a,p,I_* = Emissions of pollutant *p* from aircraft type *i* at airport *a* for time period *T* (t/T);
- *EF_p,i_* = Emission factor for pollutant *p* for aircraft type *i* (g/LTO);
- Δ*_a,i_* = Number of LTO cycles for aircraft type *i* at the airport *a* (LTO/T).

Factor 10^−6^ is applied to convert emissions from grams (g) to metric tons (t), ensuring compliance with international emission reporting standards. This activity-based approach ensures that emissions are estimated based on real-time aircraft operations, particularly within the Landing and Take-Off (LTO) cycle, which includes approach, taxi-in, taxi-out, takeoff, and climb-out up to 3000 feet. By adopting this standardized methodology, the study provides a robust and internationally recognized framework for evaluating aviation-related air pollution [23,24].

Meteorological parameters: Weather parameters, comprising monthly average temperature (temperature °C), rain (mm), average sunshine duration (INST), and maximum wind (Beaufort), were acquired from the nearby meteorological station (https://w1.meteo.gr/Gmap.cfm accessed on 1 April 2023). The automatic airport station measured all basic meteorological parameters in the area and represented the weather conditions and the respective climate data from the airport area.

The dataset consists of monthly aviation emissions and meteorological data collected for Alexandroupolis airport in 2019 and 2020. It contains the following fields: Year: the calendar year of data collection; Total Traffic: the total number of aircraft movements recorded monthly; Aircraft Type: the specific aircraft models operating during the month; Month: the corresponding month of data collection; CO_2_ (kg): the total carbon dioxide emissions from aircraft operations; NO_x_ (kg): the nitrogen oxide emissions from aircraft engines; CO (kg): the volume of carbon monoxide emissions; HC (kg): hydrocarbon emissions from aircraft fuel combustion; SO_x_ (kg): sulfur oxide emissions attributed to aviation activities; Fuel Consumption (kg): the total fuel burned during operations; PM_2.5_ (kg): fine particulate matter (PM_2.5_) emissions from aircraft operations. Meteorological parameters (temperature, rainfall, sunshine, and wind speed) are monthly averages representing prevailing weather conditions; the emissions data are based on average emission factors for different aircraft types, calculated according to monthly total aircraft traffic.

The aviation emissions dataset consists of monthly cumulative values, meaning that for each pollutant (e.g., CO_2_, NOx, PM_2.5_), the total monthly emissions are recorded based on the sum of emissions from all aircraft operations within the month. Accordingly, the meteorological data consist of monthly averages, with temperature, wind speed, precipitation, and sunshine duration averaged over the corresponding month for 2019 and 2020. This distinction ensures emissions reflect the total aviation activity while meteorological data represent prevailing atmospheric conditions. To enhance clarity, Supplementary Materials Table S1 presents a sample of the dataset used in the study, demonstrating how emissions and meteorological parameters are structured for analysis.

This research’s set of measurements consists of 168 measurements for two years, 2019 and 2020. This dataset records aviation emissions as monthly cumulative values and meteorological parameters as monthly averages. As mentioned above, limitations in data availability and the operational characteristics of the border regional airport of Alexandroupolis limited the study period to two years. Unfortunately, the recording of meteorological data before this period presents some inconsistencies, making it difficult to ensure reliable long-term data. However, the choice of this period provides the study of the impact of the pandemic crisis on air traffic in 2020, namely pre-pandemic vs. pandemic emissions. In particular, the sharp decline in aviation activity due to restrictions imposed on air travel at national and international levels provides a unique opportunity to examine pre-pandemic emissions versus the evolution of the pandemic, offering valuable insights into how operational disruptions affect air quality.

This study provides a substantial snapshot of aviation-related emissions. Future research efforts should focus on a larger dataset, e.g., 2015–2025, to decipher long-term trends, the rate of air traffic recovery, and the corresponding impacts. However, such a large-scale study would require consistent methodologies for collecting all data over many years to ensure its comparability and reliability.

To summarize, 168 measurements were catalogued for all parameters, air pollutants from aviation emissions, and meteorological variables in 2019 and 2020. Descriptive statistics and the Pearson correlation coefficient were applied to the meteorological and pollutant variables at a 0.01 confidence level, except otherwise stated.

### Data Description, Machine Learning Models, and Evaluation Metrics

**Figure 2:**
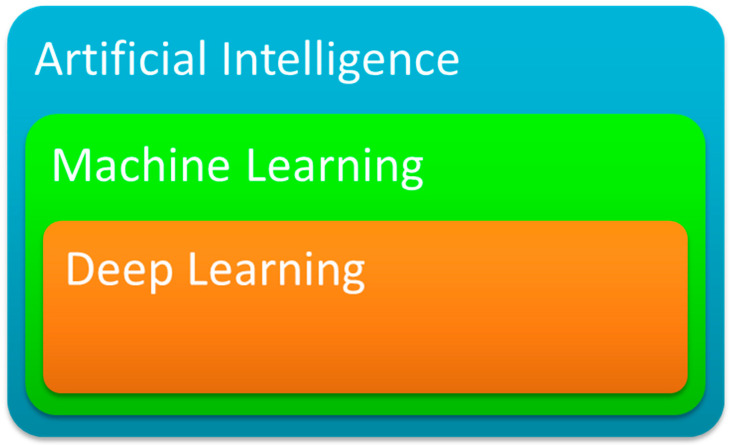
Machine learning as a subfield of Artificial Intelligence.

Machine learning, a subset of Artificial Intelligence, focuses on granting computers the capacity to acquire the skills needed to execute particular tasks without explicit human programming. It revolves around creating models that can absorb knowledge from data and subsequently use it to make informed decisions or predictions when confronted with new available data (Figure 2) [25].

Linear Regression is a straightforward choice for basic predictive tasks, performing well on high-dimensional, sparse datasets. Decision trees are non-parametric models that efficiently navigate data using simple tests and are great for nonlinear decision boundaries. In regression, an ensemble of decision trees creates a combined Gaussian distribution prediction. Gradient boosting is a powerful technique for regression, incrementally building trees while minimizing error. It excels in handling complex problems with a stepwise approach. Bayesian inference aids data analysis and learning. Fields like medicine need to assess prediction uncertainty. Neural networks can be used for Regression, offering adaptability in modelling nonlinear functions, especially in complex scenarios [26,27,28,29,30,31].

In the study proposed here, the air pollution database for the city of Alexandroupolis, Greece, was considered, and an attempt was made to predict the emissions levels of PM_2.5_ using various machine learning methods. Five basic algorithms, Bayesian Linear Regression, Boosted Decision Tree, Linear Regression, Decision Forest Regression, and Neural Network Regression, were used for regression analysis using machine learning methods. The machine learning algorithms used in this research are presented here briefly: Decision trees are non-parametric models that efficiently navigate data using simple tests and are great for nonlinear decision boundaries. In regression, an ensemble of decision trees creates a combined Gaussian distribution prediction. Gradient boosting is a powerful technique for regression, incrementally building trees while minimizing error. It excels in handling complex problems with a stepwise approach. Linear Regression is a straightforward choice for basic predictive tasks, performing well on high-dimensional, sparse datasets. Bayesian inference aids data analysis and learning. Finally, neural networks can be used for Regression, offering adaptability in modelling nonlinear functions, especially in complex scenarios (https://learn.microsoft.com/en-us/azure/machine-learning/component-reference/boosted-decision-tree-regression?view=azureml-api-2, accessed on 1 March 2024) [2,32].

**Figure 3:**
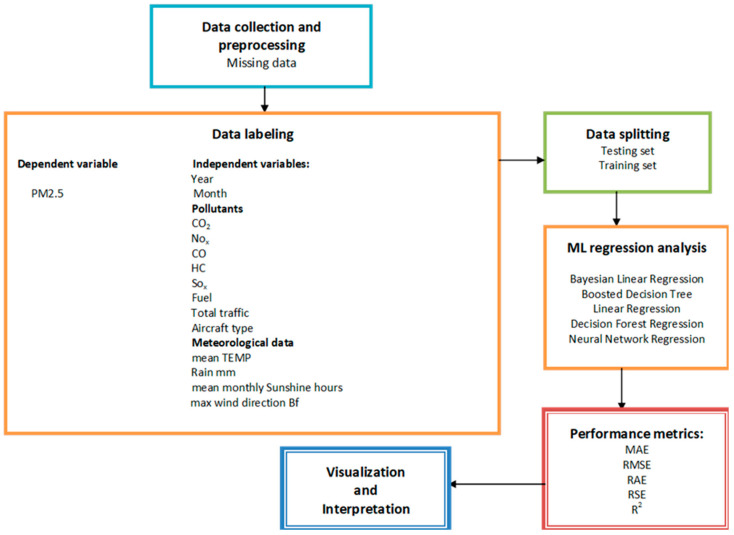
Research workflow of the proposed methodology.

Classical regression-based algorithms, especially machine learning ones like Decision Trees and Random Forest, have been widely applied in forecasting air quality levels and characteristics. Regarding predictor variables, three main categories were discerned: variables associated with pollutant concentrations, meteorological parameters, and variables about temporal and spatial characteristics [33]. In the same survey, PM_2.5_ was the most predicted pollutant among the analyzed documents. Consequently, a combination of variables from the three categories mentioned above was chosen to anticipate PM_2.5_ concentration in this research study. Figure 3 illustrates the research workflow of the proposed system.

The evaluation metrics, such as Mean Absolute Error (MAE), Root Mean Square Error (RMSE), Relative Absolute Error (RAE), Relative Squared Error (RSE), and Coefficient of Determination (R^2^), were used to assess all the machine learning methods. Evaluation metrics are valuable tools for determining the performance of machine learning models, and they can be categorized into two groups. On the one hand, range-dependent metrics are used to compare different models on the same dataset. On the other hand, percentage metrics facilitate model comparisons independently of the dataset. Some of the most commonly used metrics in the analyzed studies in regression are R^2^ and MAPE (20.1% each). RMSE/MSE and MAE are prevalent among the range-dependent metrics, appearing in 68.45% and 46.3% of the publications, respectively [34].

The robust evaluation framework of the models can be briefly explained as follows: A lower value signifies a more robust prediction ability regarding the mean absolute error. Likewise, a smaller value indicates more substantial predictive capabilities for root mean squared error. In the case of mean absolute error, better model performance is displayed by lower values. On the contrary, the coefficient of determination (R^2^) assesses the model’s predictive capacity, which is revealed to have higher values [34]. Supplementary Materials Figure S1 presents all modelling parameters extracted from the Microsoft Azure Studio Classic for regression machine learning algorithms.

Finally, it is also essential to understand the predictive ability of machine learning models, apart from their evaluation framework, through specific metrics as mentioned above. In this vein, the Permutation Importance method (PIM) was used to interpret the best machine learning model that will result [35,36]. The process’s function is simple and practically based on the fact that if a variable is essential for the model’s predictive ability, rearranging its values will significantly reduce its accuracy. On the contrary, if rearranging the values of the variable leaves the predictive ability of the model indifferent, then this variable does not significantly impact the predictive ability of the model. Furthermore, this method can be applied to any machine learning model, and thus, its application does not require retraining the model but only rearranging the input values. Consequently, the researcher is provided with a direct estimate of the importance of all features based on the modification in the model performance [37,38,39].

## 3. Results

**Figure 4:**
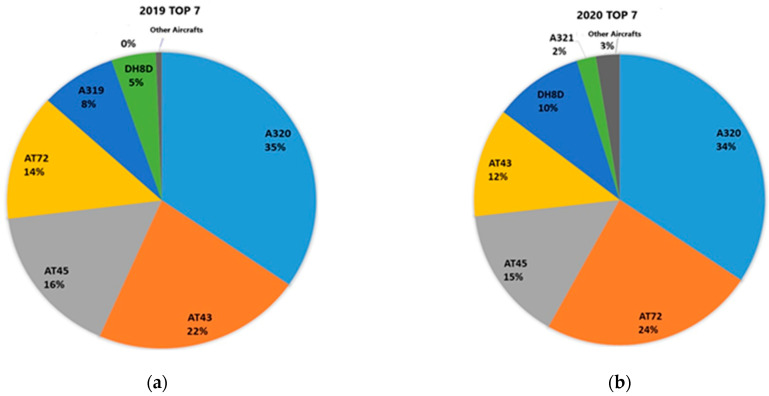
(**a**) Fleet composition in Alexandropoulis airport—2019. (**b**) Fleet composition in Alexandropoulis airport—2020. Abbreviations: A320—Airbus A320, A319—Airbus A319, AT43—ATR 42-300/320, AT45—ATR 42-500, AT72—ATR 72-200/500, DH8D—De Havilland Canada Dash 8 Q400.

As stated, all information about air traffic and fleet composition, depicted in Figure 4, was gathered from the Hellenic Civil Aviation Authority (CAA).

Figure 4 shows the aircraft that used Alexandroupolis airport in 2019 and 2020. The A320 had the largest percentage share, followed by the AT43, with 35% and 22%, respectively. The remaining aircraft types for 2019 shared percentages of 16%, 14% for the AT45 and AT 72, while the A319 and DH8D aircraft had percentages below 10%. A similar picture is presented in the percentage shares by aircraft type in 2020 at Alexandroupolis airport. Specifically, the A320 held the most significant percentage with 34%, while the AT72 share increased to 24%. The AT45 (15%) and AT43 (12%) aircraft types had slight changes compared to the previous year. The share of DH8D aircraft increased to 10%, and other aircraft accounted for 2%, indicating a slight diversification in the fleet composition.

**Figure 5:**
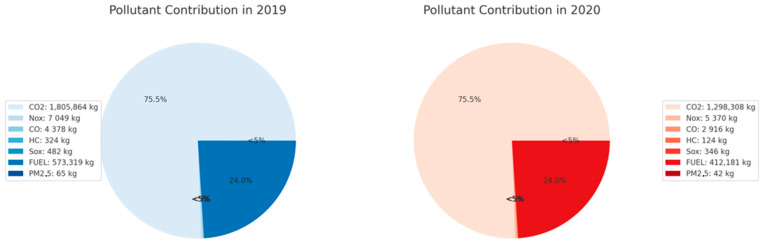
Percentage contribution of pollutants in 2019 and 2020.

Figure 5 illustrates the two pie charts representing the percentage contribution of the pollutants under study for 2019 and 2020.

In 2019, carbon dioxide had the most extensive percentage distribution, accounting for 75.5% of total emissions, followed by fuels, which accounted for 24%, and the remaining pollutants, such as NO_x_, CO, HC, SO_x_, and PM_2.5_, had more minor impacts. In 2020, the same pattern is presented. Namely, carbon dioxide represents the most significant percentage of pollutants, while the rate of fuels, although decreasing compared to 2019, still has the second percentage distribution among them.

Furthermore, although the proportions of the remaining pollutants represent approximately the exact percentages, it is evident that an overall decrease is observed, which can be attributed to the sharp reduction in air transport and activity due to the restrictive measures imposed during the pandemic crisis. The most significant decrease was observed for hydrocarbons (62.2%), followed by PM_2.5_ (35.4%), CO (33.4%), and CO_2_ (28.1%), NO_x_ (26.5%), SO_x_ (25.3%), and fuel consumption, which decreased by 22.9%. The above highlights the significant influence of air traffic volume on emission levels and emphasizes the need for further mitigation strategies to control aviation-related pollution. In conclusion, carbon dioxide is the pollutant with the most considerable percentage contribution to emissions.

**Figure 6:**
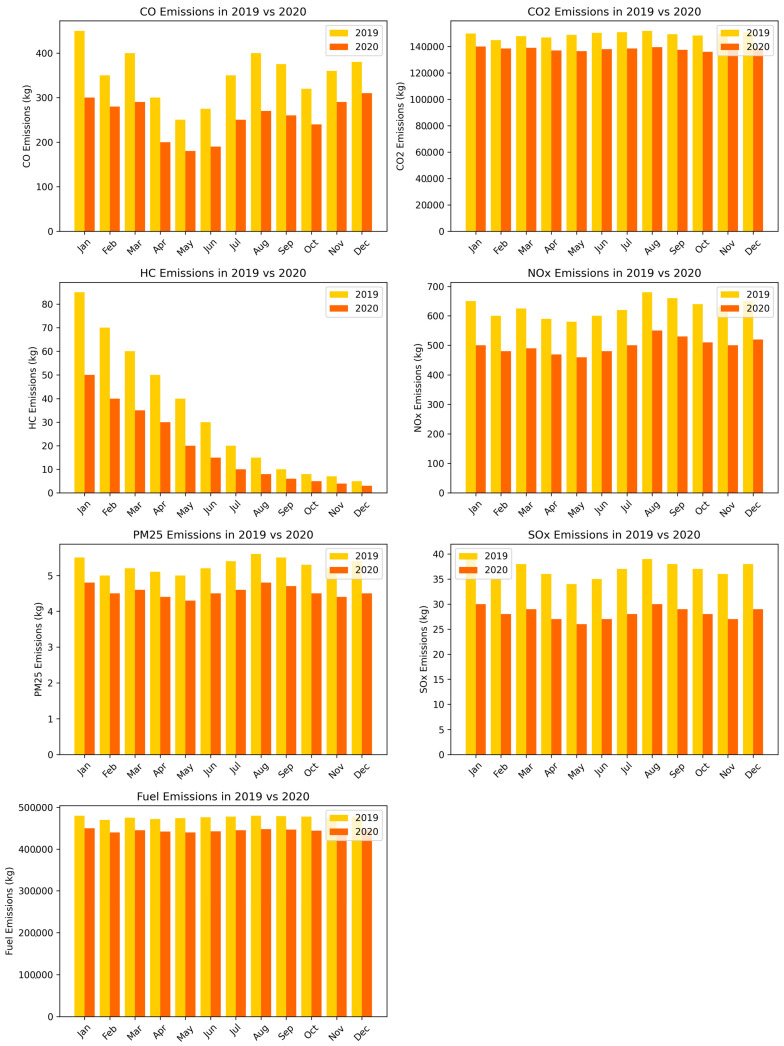
Comparison of monthly emissions for various pollutants in 2019 and 2020: CO; CO_2_; HC; NO_x_; PM_2.5_; SO_x_; fuel.

Figure 6 depicts the monthly comparison of pollutant emissions between 2019 and 2020 through seven bar graphs. Each graph describes the variation of a specific pollutant per month, with yellow representing 2019 emissions and orange representing 2020 emissions.

Figure 6 shows the monthly carbon monoxide emissions, with a lower concentration in 2020. The exact figure shows a decrease in carbon monoxide emissions in both years in 2020. Moreover, hydrocarbon emissions in 2020 are significantly lower, mainly at the beginning of the year. In Figure 6, which depicts the monthly emissions per year of nitrogen oxides, decreasing trends appear in 2020 compared to 2019. The PM_2.5_ concentrations also decreased in 2020 compared to 2019, with the difference being less pronounced than in the other pollutants. Also, monthly sulfur oxide emissions show less change, decreasing in 2020. Finally, fuel emissions also follow a downward trend in 2020, indicating a decrease in consumption. Generally speaking, pollutant emissions are lower in 2020 due to changes in activities that affect fuel combustion and gas emissions, such as restrictive measures due to the COVID-19 pandemic (Supplementary Materials Figure S2 visualizes all monthly trends of pollutant emissions in 2019 and 2020).

**Figure 7:**
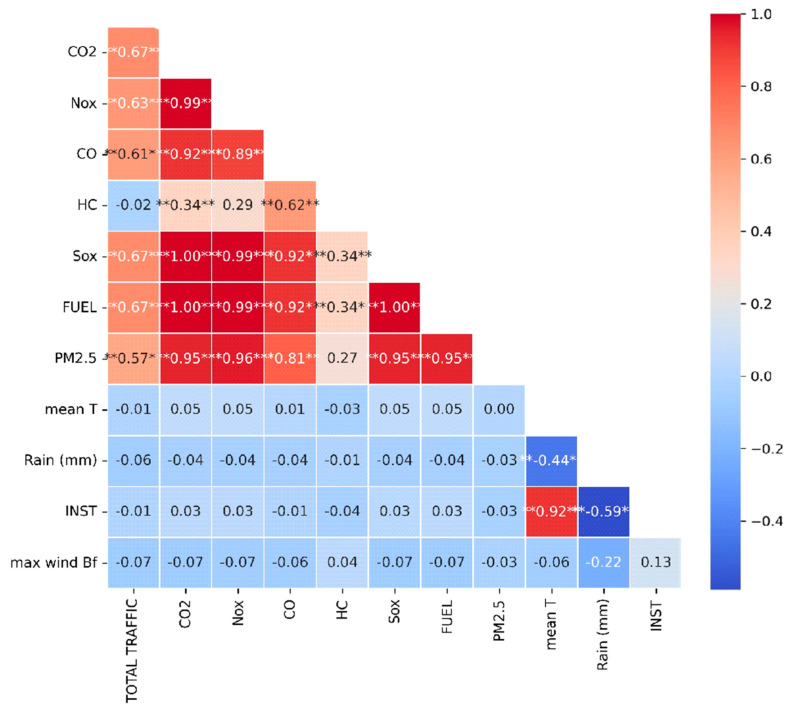
Heatmap of Pearson correlations between meteorological and pollutant variables. ** Correlation is significant at the 0.01 level (two-tailed), * moderate correlation, *** strong correlation.

Table 1 and Figure 7, respectively, give the descriptive statistics and the values of the Pearson correlation coefficient between the meteorological and pollutant variables. The correlations between meteorological parameters, the pollutant variables, and the respective descriptive statistics are presented in the table below (Table 1).

The above table shows the descriptive characteristics of the measurements for the variables that determine air pollution and the measurements of the collected meteorological parameters. It shows the range, minimum, and maximum values of the parameters, the average, the variance, the standard deviation, and the standard error of the variable’s values.

Nitrogen oxides (NO_x_) show a wide range of values and high standard deviations, indicating large fluctuations in the recorded values of their presence. Hydrocarbons (HC) and sulfur oxides (SO_x_) show variability in their concentrations, with lower values. Regarding PM_2.5_, occasional peaks in their presence are observed from the values of their descriptive statistics. Regarding fuel and carbon dioxide emissions, fuel consumption has the most extensive range (0–48904.8) and an average value of 9026.7, indicating significant fuel consumption and usage.

From the recording of meteorological parameters, the average temperature appears to have a value of 16.9 °C, and precipitation has an average value of 45.45 mm, indicating fluctuating weather conditions. The total traffic based on the recorded flights reaches an average of 16, with an average sunshine duration of 228 min. In conclusion, the high variation of pollutants such as NO_x_, CO, and CO_2_ indicates the seasonal variation of flights. Concurrently, the relatively low levels of PM_2.5_ values against the background of the fluctuation in the values of meteorological parameters (wind, rain) indicate the variability in the dispersion levels of pollutants.

Figure 7 outlines the visual representation of the Pearson correlation coefficients between different pollutant concentrations (e.g., NO_x_, CO, SO_x_, PM_2.5_, CO_2_) and meteorological parameters (e.g., mean temperature, rain precipitation, wind speed). Briefly, the red tones imply positive correlations (closer to +1), the blue tones indicate negative correlations (closer to −1), while white or light colors demonstrate weak or absent correlations between the variables.

Strong positive correlations are shown between CO_2_ and NO_x_, SO_x_ and NO_x_, and fuel with CO_2_ and NO_x_, possibly due to familiar sources of fuel combustion and air traffic emissions. Fuel consumption also contributes to carbon dioxide emissions. Finally, PM_2.5_ is strongly associated with NO_x_ and SO_x_.

Conversely, moderate correlations between total traffic, PM_2.5_, and CO_2_ are shown, as more air traffic leads to increased suspended particles and carbon dioxide emissions.

Rainfall shows negative correlations with PM_2.5_ and sunshine duration since it is known that rainfall reduces the concentrations of these particles. In conclusion, the meteorological parameters, temperature, and wind speed do not significantly affect pollutant concentrations, with the corresponding Pearson correlation coefficients ranging at levels that indicate weak or no correlation. In terms of statistical significance, airplane fuel consumption and total traffic appear to be the primary drivers of air pollution, significantly affecting the effect of emissions of the above pollutants.

**Figure 8:**
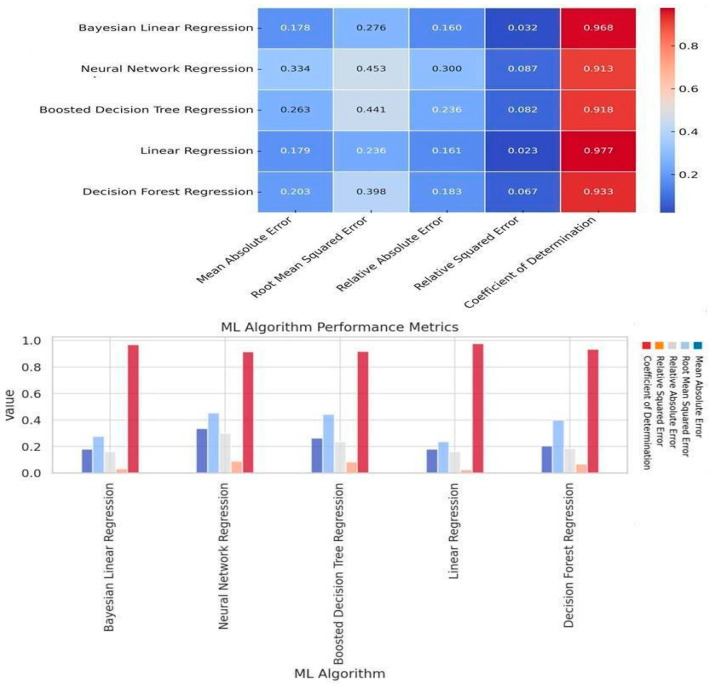
Evaluation of the regression machine learning algorithms and the performance heat map.

In Figure 8, the comparison of the proposed algorithms is represented. Bayesian Linear Regression and Linear Regression performed better than the other algorithms. These two had almost the same Coefficient of determination metric (R^2^) score. Linear Regression had the lowest value of Relative Squared Error. In contrast, the Bayesian Linear Regression algorithm had the lowest value for the metrics Mean Absolute Error, Root Squared Error and Relative Squared Error. The remaining algorithms show mixed trends in their evaluation metrics. Some excel in one metric and others in another.

The extant algorithms exhibit disparate trends in their respective evaluation metrics, manifesting prowess in distinct domains. Notably, Neural Network Regression encountered suboptimal error rate performance while attaining a commendable Coefficient of Determination value. This is evident in its elevated values across multiple error metrics, including Mean Absolute Error, Root Mean Squared Error, Relative Absolute Error, and Relative Squared Error.

Conversely, the Boosted Decision algorithm demonstrated moderate performance across most metrics, except the Coefficient of Determination, where it ranked second-lowest compared to its algorithmic counterparts. In a final analysis, the Decision Forest Regression emerged as the third-best performer among the ensemble of algorithms under consideration. This conclusion is substantiated by its superior performance in Absolute Error, Root Mean Square Error, Relative Absolute Error, Relative Mean Square Error, and Coefficient of Determination compared to the remaining machine learning algorithms, as illustrated in Figure 8.

**Figure 9:**
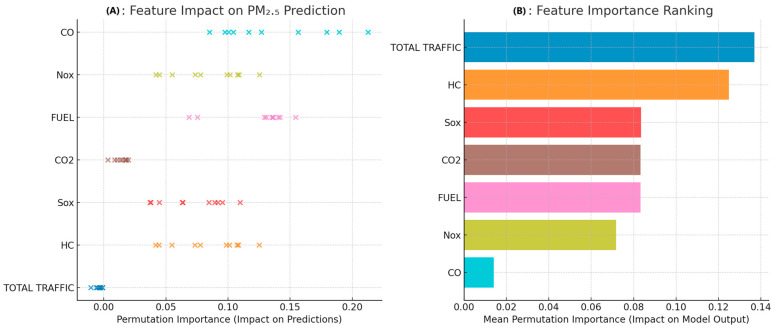
Feature importance analysis for PM_2.5_ prediction.

Figure 9 shows the effect of features on the prediction of PM_2.5_ levels for the Bayesian Linear Regression model. The analysis is based on the Permutation Importance Method, and the importance of the features is depicted in two different ways.

Panel A visualizes the features’ importance at their variation level in predicting PM_2.5_ levels. Each point shows the degree to which it contributes to the model. Thus, it is noted that carbon monoxide, NO_x_, FUEL, CO_2_, SO_x_, HC, and TOTAL TRAFFIC concentrations have different levels of influence. The TOTAL TRAFFIC feature shows the most negligible dispersion, in contrast to CO and NO_x_, which show the most considerable variability. Figure 9B shows the mean value importance of the features. TOTAL TRAFFIC emerges as the most critical factor influencing the prediction of PM_2.5_ levels, followed by the concentrations of HC, SO_x_, CO_2_, and FUEL pollutants. On the contrary, CO shows minor importance, which demonstrates that it is not a critical factor for the change in PM_2.5_ levels.

## 4. Discussion

The European Green Deal prioritizes addressing air pollution, recognizing its critical impact on public health and the environment. Proactive measures aim to pave the way for all European residents’ healthier and cleaner future. The risks posed by air pollution are severe, contributing significantly to respiratory illnesses, cardiovascular complications, and premature mortality. To combat this pressing issue, the European strategy revolves around comprehensive actions, including reducing transport, industry, and agriculture emissions [40]. In addition, air quality has begun to be investigated in terms of its impact on mental disorders, with studies attempting to elucidate the role of PM_2.5_, for example, concerning the development of depression, schizophrenia, anxiety, and bipolar disorder [41].

Furthermore, it extends to enforcing stringent air quality standards, advocating for cleaner technologies, and promoting sustainable practices across various sectors. Moreover, the European Green Deal underscores the significance of collaborating internationally and forging partnerships fostering a unified global effort against air pollution (https://ec.europa.eu/commission/presscorner/detail/en/ip_22_6278 (Last accessed 24 December 2023), https://environment.ec.europa.eu/topics/air_en (Last accessed 24 December 2023)).

In the present study, the Bayesian and Linear Regression models yielded high metric performances to predict PM_2.5_ pollutants related to aviation emissions, with R^2^ of 0.96 and 0.97, respectively. This shows high accuracy when considering the concentration of other pollutants and meteorological factors.

Also, the above prediction models effectively captured the impact of aviation activity NO_x_ and CO_2_ emissions, with Pearson correlation coefficients of 0.92 and 0.89, respectively (Figure 7). This highlights the critical role of aviation activity and the corresponding fuel consumption in the concentration of these pollutants. This finding also aligns with the existing literature on the impact of aviation activity on the concentration levels of various pollutants [42,43,44].

It should also be emphasized that the predictive ability of the models is captured in the right direction with the actual measurements of a sharp reduction in emissions in 2020, the year of the start of the pandemic crisis. Specifically, the 28.1% reductions for CO_2_ and 26.5% for NO_x_ (Figure 5) also reflect the actual picture of the reductions resulting from the corresponding air traffic reduction during this period. Other studies which evaluated the impact of the pandemic on air activity and air quality have confirmed such a pattern [45,46].

The impact on air quality due to lockdown restrictions has been observed, and concentrations of air pollutants have decreased significantly during the pandemic, mainly due to reduced anthropogenic activities. In Greece, a significant drop in urban air pollution has also been reported, for example, a reduction in NO emissions of up to 78% in urban stations and by 45% at Athens International Airport, while NO_2_ levels decreased by 73% in the two largest cities of Greece, Athens and Thessaloniki. This decrease is also due to the general reduction in aviation activity and, by extension, emissions. Lastly, it was observed that pollutants such as NO_2_ showed a sharp decrease. In contrast, pollutants such as PM_2.5_ and PM_10_ showed more variable trends influenced by meteorological variables such as wind dispersion and dust transport [47,48].

Despite machine learning models’ high predictive ability, the models do not take into account small deviations that should be attributed to factors such as meteorological fluctuations and local pollution sources. These are generally the recommendations for improving air forecast models, namely incorporating more variables that reflect weather conditions [49,50,51].

Artificial intelligence models for forecasting environmental pollution are a previously introduced concept. Investigations into employing artificial intelligence in the context of atmospheric pollution have experienced a notable surge since 2017. Within the domain of air pollution, machine learning models, with a specific emphasis on regression techniques, stand out as widely adopted approaches for scrutinizing and deciphering the distributions of air pollutants, mainly when focusing on PM_2.5_ concentration and its implications for public health [52].

Another study [53] compared several algorithms (MLR, KNN, M5P, RF, SVM, or MLP) to predict various pollutant concentrations in Valencia, Spain. Notably, RF achieved the highest accuracy [53]. Ameer et al. (2019) performed a similar comparison involving four models (RF, DT, MLP, Boosting) to predict PM_2.5_ levels in several Chinese cities, with RF demonstrating superior accuracy [54]. Li et al. (2019) pitted Logistic Regression against RF for forecasting AQI in California, and RF emerged as the more accurate predictor [55]. Pasupuleti et al. (2020) considered three algorithms (RF, DT, MLR) to forecast the concentration of various pollutants in Spain, with RF again showing the highest accuracy [56].

In a different context, Kaur Bamrah et al. (2020) compared various regressor methods (MLP, RF, DT, and SVR) for predicting AQI in India, incorporating terrain features [57]. In these studies, RF consistently achieved the highest accuracy. Yarragunta et al. (2021) compared six regression algorithms (DT, KNN, SVR, MLR, RF, and Naive Bayes) to predict AQI in Delhi, and the DT algorithm secured the highest accuracy in this particular case [58]. Chakradhar Reddy et al. (2021) conducted a comprehensive comparison of six supervised ML models (LR, RF, DT, SVR, KNN, and Naive Bayes) for forecasting AQI in New Delhi [58], and the results indicated that DT achieved notably high accuracy, approaching 100% [59,60].

In this current research, Bayesian Linear Regression and Linear Regression algorithms were the most accurate. Particulate matter concentrations, specifically PM_2.5_, are predominantly influenced by pollution emissions and prevailing weather conditions [61]. Over four years, Kou et al. (2021) [61] scrutinized the meteorological impact on PM_2.5_-related air quality in China between 2016 and 2019, utilizing a high-resolution atmospheric composition reanalysis dataset [62]. The correlation between weather patterns and air quality was further investigated. The results indicated that, in tandem with China’s stringent enforcement of its clean air policy from 2016 to 2019, meteorological conditions played a constructive role in enhancing air quality [20]. In a separate investigation, Alpan and Sekeroglu (2020) employed machine learning algorithms to predict six pollutant levels, integrating meteorological data such as precipitation and temperature [62]. The Random Forest algorithm demonstrated a high predictive capability across two distinct datasets. The authors asserted that accurate forecasts of pollutant concentrations could be achieved solely by utilizing meteorological data [63].

Ambient air pollution is a significant global health concern, contributing to over 3 million premature deaths worldwide, with Low- and Middle-Income Countries (LMICs) bearing the majority of this burden. In these countries, facing air pollution levels classified as public health hazards, megacities resort to emergency measures like red alerts and vehicle-rationing interventions (VRIs). Even during interventions, both cities experienced increased cardiopulmonary mortality, emphasizing the need for short- and long-term strategies to manage the health impacts of air pollution [64].

Analyzing the dynamics behind fine particulate matter (PM_2.5_) and ozone (O_3_) pollution across key regions in China, extensive studies employed the Weather Research and Forecasting/Community Multiscale Air Quality (WRF/CMAQ) system from 2013 to 2019. The model demonstrated high accuracy, evaluating against observed pollutants in significant areas like the North China Plain, Yangtze River Delta, Pearl River Delta, Chengyu Basin, and Fenwei Plain, slightly overestimating PM_2.5_ in one region. Notably, nitrate (NO_3_^−^) and ammonium (NH_4_^+^) emerged as vital PM_2.5_ components in heavily polluted zones. This analysis highlighted negative correlations between PM_2.5_ and O_3_ in most areas, underscoring the model’s ability to simulate China’s long-term air quality trends, which is crucial for effective emission control strategies [65].

Furthermore, understanding pollutant emission sources is crucial for effective mitigation. Air quality data from urban, suburban, industrial, and rural areas in Jining, Shandong Province, China, were compared for characteristics and health risks associated with air pollutants. Variances in PM_2.5_, PM_10_, SO_2_, NO_2_, and CO concentrations between 2017 and 2018 were observed, with O_3_ concentrations increasing. Functional areas exhibited similar seasonal variations and diurnal patterns, with O_3_ contributing significantly to exposure excess risks (ERs). Premature deaths attributable to air pollutants were calculated, highlighting O_3_ as the significant contributor. Pollution transport from industrial areas to urban and suburban regions played a crucial role in determining air quality, emphasizing urgent measures to reduce O_3_ pollution, particularly considering the prevalent ozone formation regime in industrial areas [66].

Air pollution and climate change exhibit intricate interdependencies, where climate fluctuations impact air pollution dynamics and vice versa. This relationship is complex, with emissions of air pollutants affecting climate through radiative forcing and climate changes altering the physical, chemical, and biological processes linked to air pollution. High-pressure weather conditions tend to be associated with elevated PM_2.5_ and O_3_ levels. Seasonally, PM_2.5_ concentrations are higher during the winter, while O_3_ concentrations are higher during the summer [67]. Uncertainties persist despite recognizing these interactions, requiring deeper insights to comprehend their mechanisms and consequences. Additionally, the co-emission of greenhouse gases (GHGs) with air pollutants suggests the potential for synergistic mitigation strategies. Yet, the existing literature needs an in-depth understanding of these co-benefits [68].

Notably, research has shown a link between long-term exposure to PM_2.5_ and child mortality, with studies confirming this pattern in countries in Asia, Africa, and Latin America and an additional decline in living standards due to air pollution [69,70,71,72]. Given the impact of air pollution on the levels and severity of respiratory diseases, especially among elders and children, machine learning methods were applied to link air pollutants, seasonal variation, and climate data. A study in Taizhou, China, utilized various machine-learning models, including Linear Regression, Random Forest (RF), AdaBoost, and Neural Networks, to investigate the relationship between air pollutant concentrations and pediatric respiratory diseases. The findings reveal significant seasonal fluctuations in both the numbers of pediatric respiratory outpatients and the concentrations of air pollutants. NO_2_, CO, particulate matter (PM_10_ and PM_2.5_), and outpatient numbers peak during the winter, indicating a substantial impact of air pollution on pediatric respiratory diseases. Regression models demonstrate that ML methods capture clinic visit trends and turning points, with nonlinear models outperforming their linear counterparts. Notably, the RF model emerged as the most effective [73].

The burden of air pollution is disproportionately related to factors such as age and gender. An additional study showed that short-term exposure to air pollutants, mainly gaseous pollutants such as NO_2_ and CO, is linked with an escalated risk of hospital visits for AD in a city in southern China with low pollution concentrations. The age group of women between the ages of 45 and 64 seems to be most affected, providing evidence that the level of air pollution may be a risk factor, even for anxiety disorders [74].

The influence of meteorological factors such as wind is significant for the dispersion of pollutants, specifically PM_2.5_, and air masses at a local level. The fluctuation of air masses at a seasonal level can also affect the transport of these particles and alter the level of air quality [75]. In our research, four meteorological parameters were considered when estimating air quality in Alexandroupolis. Meteorology, atmospheric reactivity, and emissions at the regional level are, among other factors, the most contributing factors in the temporal variability of PM_2.5_ concentration and air quality, as revealed in a study that applied statistical methods to consider PM_2.5_ daily measures and meteorological parameters in India [26]. The airport in Alexandroupolis primarily caters to domestic flights and experienced minimal alterations in flight frequency and fleet composition from 2019 to 2020. Nevertheless, in both years, there was a notable rise in emission concentrations—including fuel, NO_x_, CO, HC, SO_x_, and PM—during the summer and New Year seasons, coinciding with increased travel activity.

Temperature is also essential to CO emissions, as low temperatures reduce aircraft fuel evaporation due to inefficient combustion, resulting in increased carbon monoxide emissions. Humidity was also positively correlated with the above aircraft exhaust emissions [74,75,76].

Comparing the present study with other approaches to emissions and pollutants at other airports in Greece, with a different methodological approach, it was found that NO_2_ concentrations exceeded regulatory limits by almost 30% of the cases under specific meteorological conditions. In the same study, although the PM_10_ and SO_2_ concentration levels were within limits for air quality standards, in the present study, the maximum value recorded for PM_2.5_ was 5.6 and for SO_x_ was 41, indicating that in smaller and regional airports, there is a different dynamic and distribution of these pollutants, possibly due to, among other things, local emissions from aircraft, ground vehicles. Finally, although the approach in the above study involves static dispersion modelling, the machine learning models here offer a slight advantage because they consider the real-time estimation of pollutant levels based on changing meteorological conditions and airport activity. This dynamic capability benefits proactive air quality management, while dispersion models mainly provide ex-post emission assessments [77,78].

In closing, we will also refer to a study that concerns the impact of lockdowns during the pandemic crisis at the two largest airports in Greece. What was observed is that at the airport of the capital of Greece, Athens, NO_2_ and CO concentrations decreased by 45% and 30%, respectively, highlighting the dominant role of air transport and aviation in urban air pollution. Although there are no data yet for Alexandroupolis airport to make a comparison of pollutant concentrations before, during, and immediately after the pandemic crisis, we can speculate that other phenomena, such as extreme weather phenomena such as the dust transport observed in Greece and agro-industrial activities around the airport, may also contribute to the sources of atmospheric air pollution at airports and PM [47,48,79,80].

## 5. Limitations

The present study on applying machine learning algorithms for predicting PM_2.5_ concentrations in Alexandroupolis bears certain methodological constraints. Notably, the temporal scope of the investigation, limited to a relatively abbreviated period, suggests the potential for enriched insights through an extension across multiple years. A refinement of the study’s comprehensiveness could be achieved by incorporating a broader array of meteorological variables and epidemiologically relevant medical data, thereby enhancing the contextual richness of the predictive modelling.

Despite these acknowledged limitations, this study represents a seminal contribution as one of the initial endeavors to systematically examine the interplay between air pollutant concentrations, meteorological parameters, and the influence of aircraft flights. Understanding these intricate relationships is pivotal in devising effective mitigation measures and policies.

## 6. Conclusions

This work advances our understanding of air quality and pollution dynamics. It establishes a precedent for subsequent empirical inquiries in Greece, laying the groundwork for more comprehensive studies in the emerging field of air pollution research. The perspectives from this investigation contribute to the growing body of knowledge at the intersection of environmental science, air pollution, epidemiology, meteorology, climate change, and public health.

Analysis and prediction of air pollution levels at airports are essential topics in atmospheric and environmental research due to air pollution’s impact on human health and quality of life. Predicting the maximum concentration of the above parameters in the atmospheric air is of great importance for controlling and improving the quality of the atmosphere. The ultimate goal is the sustainable development of the airport region concerning public health issues.

Due to the increased choice of aircraft as a means of transport and the growth of the aviation industry, aircraft emissions are skyrocketing to be ignored, and policymakers, including environmental, legal, regulatory, and public health aspects, may propose practical strategies for minimizing the effects of global warming and climate changes on health.

This research advocates for a dynamic approach to deploying effective policies and strategies to underscore the imperative of sustaining prevention and control measures for air pollution in airport environments. By addressing the specific challenges associated with air quality management in airports, the study aims to contribute to developing comprehensive and adaptable measures for controlling and mitigating pollution in these settings.

## Figures and Tables

**Table 1:** Descriptive statistics of the meteorological and pollutant variables.

Parameter **	N	Range	Minimum	Maximum	Mean	Std. Error	Std. Deviation	Variance
NO_x_	168	602.9	1.9	604.8	103.8	11.5	146.4	21,445.3
CO	168	415.7	2.3	418	82.8	6.5	83.4	6967.6
HC	168	87.9	0.1	88.0	8.1	1.26	12.75	162.79
SO_x_	168	40.9	0.2	41.1	7.58	0.77	9.87	97.55
Fuel	168	48,904.8	199.4	48,705.4	9026.7	925.8	11,748.1	138,017,958.8
PM_2.5_	168	5.52	0.07	5.6	1.69	0.22	1.84	3.4
CO_2_	168	153,411.2	628	154,039.2	28,433.02	2916.33	37,004.19	1,369,310,238.3
Total Traffic	168	55.0	1	56	16.12	1.2	15.04	226.43
Mean T (C^0^)	168	22.8	5.7	28.5	16.9	0.6	7.6	58.4
Rain (mm)	168	131.0	0.0	131.0	45.45	2.75	35.73	1276.7
INST (min)	168	316.0	76.0	392.0	228.0	7.21	93.48	8740.1
Max Wind (Bf)	168	3.0	3.0	6.0	4.2	0.04	0.57	0.33

** Parameters: Fuel, NO_x_, CO_x_, SO_x_, PM_2.5_, SO_x_: Total traffic emissions [Kg].

## Data Availability

The data presented in this study are available on request from the corresponding author.

## Supplementary Materials

The following supporting information can be downloaded at https://www.mdpi.com/article/10.3390/toxics13030217/s1: Figure S1. Modeling parameters are extracted from the Microsoft Azure Studio Classic for regression machine learning algorithms. Figure S2. Monthly trends of pollutant emissions in 2019 and 2020: (a) CO; (b) CO_2_; (c) HC; (d) NO_x_; (e) PM_2.5_; (f) SO_x_; (g) Fuel. Table S1. Dataset sample.
