# Supplementary material for: Assessing the Impact of Aviation Emissions on Air Quality at a Regional Greek Airport Using Machine Learning
Source: Toxics. 2025 Mar 16;13(3):217. doi: 10.3390/toxics13030217 (PMC11945904; doi:10.3390/toxics13030217)
Supplement: Supplementary file 1 [file toxics-13-00217-s001.zip › toxics-3510383-supplementary.pdf]

Supplementary Material

Article

Assessing the Impact of Aviation Emissions on Air Quality at a Regional Greek Airport Using Machine Learning

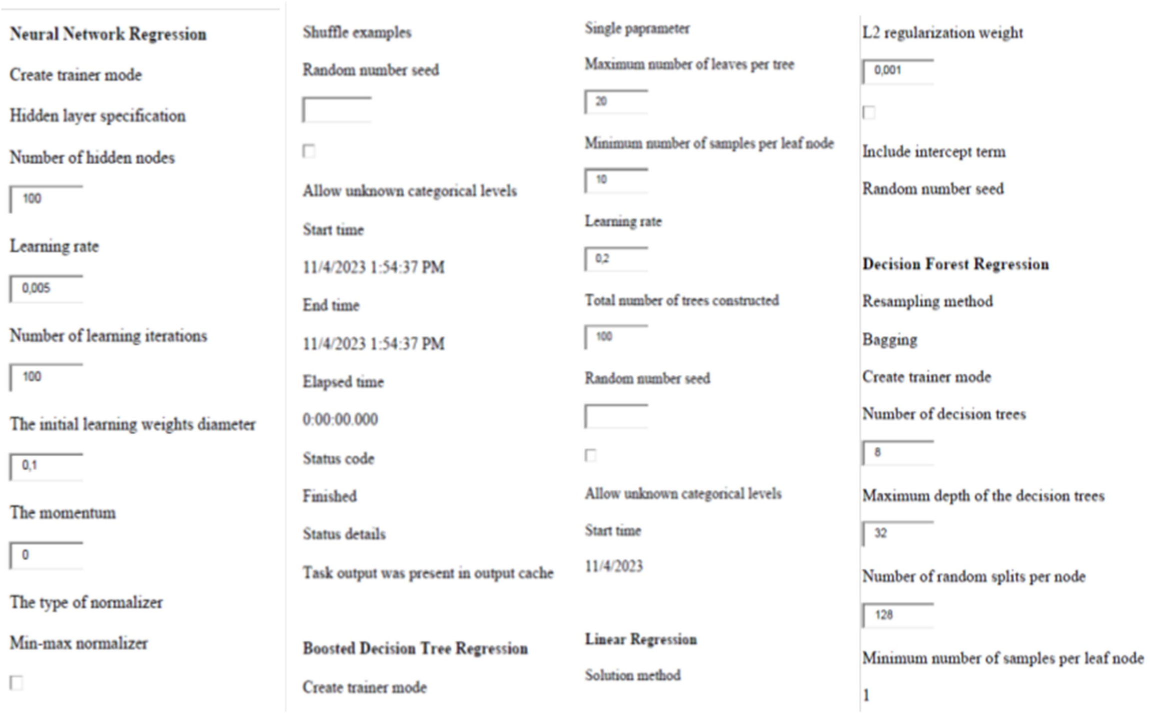

Supplementary Material-Figure S1. Modelling parameters as extracted from the Microsoft Azure Studio Classic for Regression machine learning algorithms.

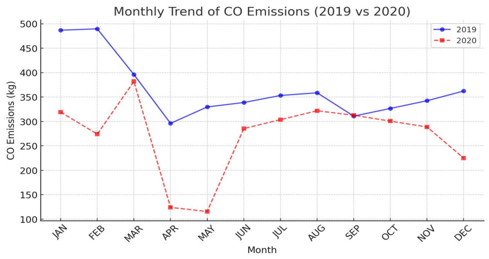

(a)

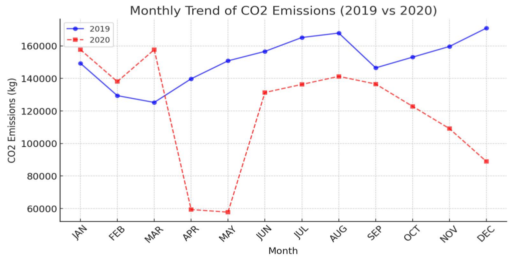

(b)

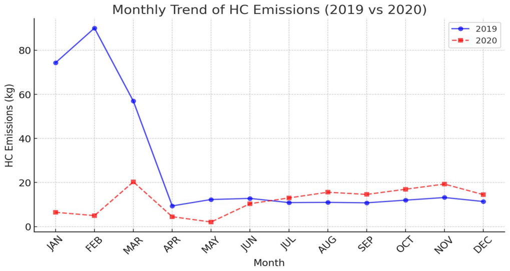

(c)

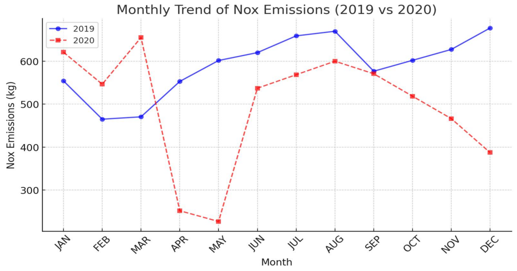

(d)

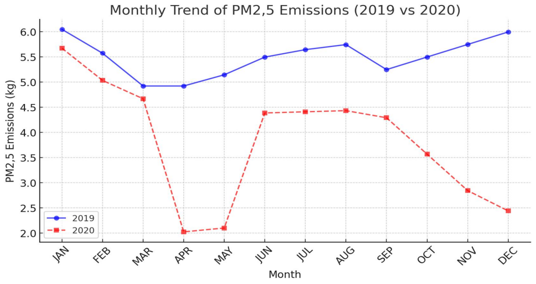

(e)

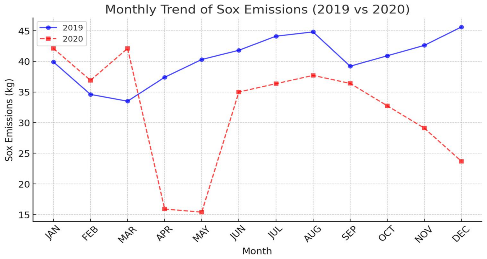

(f)

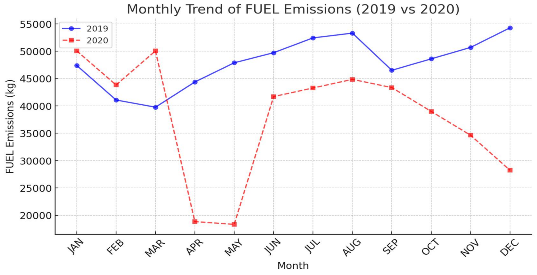

(g)

Supplementary Material-Figure S2. Monthly Trends of Pollutant Emissions in 2019 and 2020: (**a**) CO; (**b**) CO_2; (**c**) HC; (**d**) NO_x; (**e**) $PM_{2.5}$; (**f**) SO_x; (**g**) Fuel.

Supplementary Material-Table S1. Dataset sample.

year	TOTAL TRAFFIC	aircraft type	month	CO_2	No_x	CO	HC	So_x	FUEL	$PM_{2,5}$	mean TEMP	Rain (mm)	INST	max wd Bf
**2019**	24	A320	JAN	66016,8	259,2	132	2,4	17,6	20959,2	2,4	6	122	76	4
**2019**	11	A320	FEB	30257,7	118,8	60,5	1,1	8,1	9606,3	1,1	6,7	11	121	5
**2019**	21	A320	MAR	57764,7	226,8	115,5	2,1	15,4	18339,3	2,1	11,1	10	242	5
**2019**	46	A320	APR	126532,2	496,8	253	4,6	33,8	40171,8	4,6	13,4	67	212	4
**2019**	46	A320	MAY	126532,2	496,8	253	4,6	33,8	40171,8	4,6	19,4	47	269	4
**2019**	50	A320	JUN	137535	540	275	5	36,7	43665	5	26,1	44	324	4
**2019**	52	A320	JUL	143036,4	561,6	286	5,2	38,2	45411,6	5,2	26,6	46	351	4
**2019**	53	A320	AUG	145787,1	572,4	291,5	5,3	38,9	46284,9	5,3	28,2	4	367	4
**2019**	49	A320	SEP	134784,3	529,2	269,5	4,9	36	42791,7	4,9	23,2	10	284	4
**2019**	45	A320	OKT	123781,5	486	247,5	4,5	33	39298,5	4,5	17,4	95	221	4
**2019**	53	A320	NOV	145787,1	572,4	291,5	5,3	38,9	46284,9	5,3	15,5	66	111	4
**2019**	56	A320	DEC	154039,2	604,8	308	5,6	41,1	48705,4	5,6	8,9	50	98	4
